# The hierarchically mechanistic mind: A free-energy formulation of the human psyche

**DOI:** 10.1016/j.plrev.2018.10.002

**Published:** 2019-12

**Authors:** Paul B. Badcock, Karl J. Friston, Maxwell J.D. Ramstead

**Affiliations:** a Centre for Youth Mental Health, The University of Melbourne, Melbourne, 3052, Australia; b Melbourne School of Psychological Sciences, The University of Melbourne, Melbourne, 3010, Australia; c Orygen, the National Centre of Excellence in Youth Mental Health, Melbourne, 3052, Australia; d Wellcome Trust Centre for Neuroimaging, University College London, London, WC1N3BG, UK; e Department of Philosophy, McGill University, Montreal, Quebec, H3A 2T7, Canada; f Division of Social and Transcultural Psychiatry, Department of Psychiatry, McGill University, Montreal, Quebec, H3A 1A1, Canada

**Keywords:** Active inference, Evolutionary systems theory, Hierarchically mechanistic mind, Free-energy principle, Neuroscience, Psychology

## Abstract

This article presents a unifying theory of the embodied, situated human brain called the Hierarchically Mechanistic Mind (HMM). The HMM describes the brain as a complex adaptive system that actively minimises the decay of our sensory and physical states by producing self-fulfilling action-perception cycles via dynamical interactions between hierarchically organised neurocognitive mechanisms. This theory synthesises the free-energy principle (FEP) in neuroscience with an evolutionary systems theory of psychology that explains our brains, minds, and behaviour by appealing to Tinbergen's four questions: *adaptation*, *phylogeny*, *ontogeny*, and *mechanism*. After leveraging the FEP to formally define the HMM across different spatiotemporal scales, we conclude by exploring its implications for theorising and research in the sciences of the mind and behaviour.

## Highlights

• We present an interdisciplinary theory of the embodied, situated human brain called the Hierarchically Mechanistic Mind (HMM).
• We describe the HMM as a model of neural architecture.
• We explore how the HMM synthesises the free-energy principle in neuroscience with an evolutionary systems theory of psychology.
• We translate our model into a new heuristic for theorising and research in neuroscience and psychology.

***Life is poised on the edge of chaos*.**

Stuart Kauffman

The aim of our review is to unify dominant schools of thought spanning neuroscience and psychology by presenting a new theory of the human brain called the *hierarchically mechanistic mind* (HMM). Originally proposed to resolve paradigmatic divisions within psychology [1], the HMM offers an integrative perspective of the brain, cognition and behaviour that has since been leveraged to explain our species-typical capacity for depression [2], and to exemplify a new, transdisciplinary approach to the study of living systems called *variational neuroethology* [3], [4]. The HMM defines the embodied, situated brain as a complex adaptive system that actively minimises the entropy (i.e., the spread or decay) of human sensory and physical states by generating action-perception cycles that emerge from dynamic interactions between hierarchically organised neurocognitive mechanisms.

The HMM leverages evolutionary systems theory^1^ (EST) to bridge two complementary perspectives on the brain. First, it subsumes the *free-energy principle* (FEP) in neuroscience and biophysics to provide a biologically plausible, mathematical formulation of the evolution, development, form, and function of the brain [14], [15], [16]. Second, it follows an *EST of psychology* by recognising that neural structure and function arise from a hierarchy of causal mechanisms that shape the brain-body-environment system over different timescales [1], [2]. According to this perspective, human neural dynamics can only be understood by considering the broader context of our evolution, enculturation, development, embodiment, and behaviour. After describing the architectural claim that underpins the HMM, we consider these two perspectives in turn, before bringing them together with a formal definition of multiscale neural dynamics. We conclude by considering the implications of our model for theorising and research in neuroscience and psychology.

In a nutshell, the HMM rests on two cardinal elements: an EST of human cognition and behaviour that draws on four intersecting levels of explanation in psychology; and a mathematical formulation of multiscale neural dynamics based on the FEP. Our central claim is that the FEP and EST of psychology reflect two sides of the same coin – the former furnishes a non-substantive *formal theory* of neural structure, function, and dynamics; the latter affords a substantive *evolutionary theory* that can explain the particular manifestations of the FEP observed in *Homo sapiens*. By leveraging theories, frameworks, and methods originally drawn from physics and biology (which also yield EST and the FEP; [3], [4]), the HMM synthesises psychology and neuroscience with a systematic framework to formulate multilevel models of the extraordinary nexus between the brain, our minds and behaviour.

## 1 The HMM as a model of neural structure

The HMM rests on the *architectural* claim that the human brain is a hierarchically organised system of neurocognitive mechanisms that interact in a dynamic, reciprocal fashion. Under this view, the lowest or most peripheral levels of the cortical hierarchy comprise relatively *segregated*, highly specialised neural mechanisms responsible for sensorimotor processing (‘domain-specific’ systems), while its higher, deeper or more central layers consist of developmentally plastic, highly *integrated* (‘domain-general’) mechanisms. The latter are widely distributed subsystems that respond flexibly to input received from multiple lower levels, feed information downstream for further processing, and underlie the executive cognitive functions unique to humans [1].

There are two important distinctions here. First, although there are many interpretations of the neural hierarchy, here we refer to a *fractal* or *nested modular hierarchy*, which entails the repeated encapsulation of smaller (neuronal) elements in larger ones across different spatial, temporal, topological, and functional neural scales (i.e., ‘modules within modules’; [17], [18], [19]). The second is that *neurocognitive mechanism* is defined as a neural subsystem that operates at any spatiotemporal scale, ranging from a particular neuronal population through to macroscopic brain regions. Such mechanisms involve a dynamic, bidirectional relationship between specialised functional processing mediated by dense, short-range connections intrinsic to that scale (i.e., its local integration); and their global (functional) integration with other neural subsystems via relatively sparse, long-range (e.g., extrinsic cortico-cortical) connections [20]).^2^ Accordingly, the HMM implies a complementary relationship between *functional segregation* and *integration*: all neurocognitive mechanisms involve a sub-population of cells that have a common, specialised function, but they are also functionally integrated because of their distal connections with other subsystems [20], [30]. At the same time, it also recognises that some neural subsystems will be more integrated than others.

**Fig. 1:**
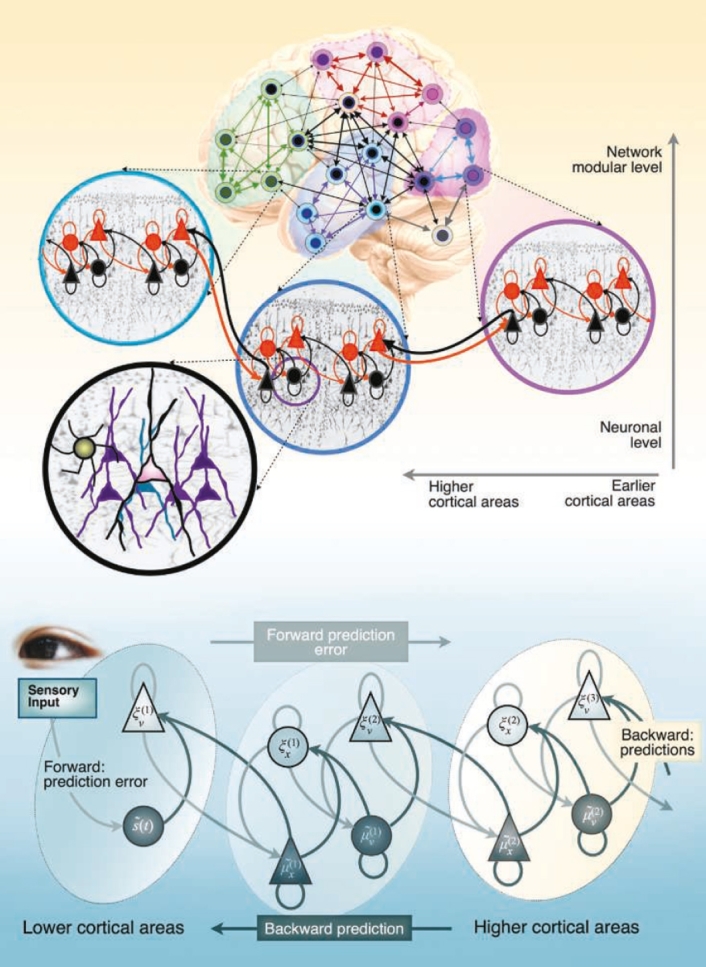
*The hierarchical organisation of neural networks*. Global brain function (i.e., cognition) can be described as the global integration of local (i.e., segregated) neuronal operations that underpin hierarchical message passing among cortical regions. Global integration is greatly facilitated by the hierarchical organisation of neural networks into (relatively modular) neurocognitive mechanisms. In network neuroscience, a neural network is modelled in terms of nodes and their connections, which are called edges. A node is defined as an integrated unit within a network. In a fractal or modular hierarchy, each node also comprises a smaller network of nodes that interact among themselves at a lower nested level. In the brain, this fractal, encapsulated hierarchy extends from neurons and macrocolumns, through to macroscopic brain regions and distributed neural networks. According to predictive coding theory, superficial pyramidal cells compare expectations at each level with top-down predictions from deep pyramidal cells at higher levels, while neuromodulatory gating or gain control of superficial pyramidal cells determines their influence on the implicit belief updating in higher hierarchical levels. Reproduced from [28].

The type of neural architecture described here echoes a growing consensus that human cognition and behaviour emerge from the integrated dynamics of hierarchical networks of (*functionally segregated* and *differentially integrated*) neural processing mechanisms [20], [25], [31], [32], [33], [34], [35], [36], [37], [38], [39], [40]. There is nothing controversial about this claim. The idea that the brain exhibits a hierarchical structure that progresses from relatively ‘domain-specific’ systems through to highly integrated, ‘domain-general’ regions is far from new, having long been recognised by influential perspectives such as global neuronal workspace theory [41], [42] and the dual process theory of reasoning [43], [44]. More recently, sophisticated structural and functional imaging studies in network neuroscience have furnished extensive evidence that human cortical networks exhibit a nested, fractal-like structure; extending from cellular microcircuits in cortical columns at the lowest level, to cortical areas at intermediate levels, through to distributed clusters of highly interconnected brain regions at the global level [45], [46], [47]; see Fig. 1a.^3^ Notably, a hierarchical neural structure is also central to the theory of *predictive processing*, an increasingly popular scheme that describes the brain as a Bayesian ‘inference machine’ that minimises discrepancies between incoming sensory inputs and top-town predictions (see Fig. 1b). Since the literature on the brain's hierarchical organisation has already been reviewed elsewhere (e.g., [18], [21], [31], [33], [49], [50]), we will not dwell on it here. Instead, we will now concentrate on the more contentious issue of *why* the brain is structured in this way.

## 2 The variational free-energy formulation

The FEP is a mathematical postulate that draws from statistical thermodynamics and machine learning to explain how living systems maintain their physical integrity by revisiting a small number of characteristic, phenotypic states [15]. It rests on the elegant premise that biotic agents actively reduce the entropy (i.e., the decay or dispersion) of their sensory and phenotypic states by minimising their *variational free-energy*. Technically, variational free-energy is an information theoretic quantity that bounds or limits (by being greater than) the *entropy* of a brain's sensations or sensory samples from the environment. In this context, entropy is a measure of information that refers to the long-term average of *surprise*: a statistical measure of the probability (technically, the negative log probability) of sensory samples sampled by an agent.^4^

The FEP stems from the idea that living systems can be distinguished from other self-organising systems because they actively avoid deleterious phase-transitions by bounding the entropy of their sensory and physical states – under the FEP, to be alive simply means to revisit a bounded set of states with a high probability [5], [52]. Here, a deleterious phase transition is cast as a *surprising* one (i.e., a low probability event, given that the creature in question continues to survive). Because the repertoire of states an organism occupies is limited, the probability density over these states must have low entropy (i.e., they are found in characteristic, unsurprising states). Heuristically, we can think of the expectations of an organism as having an evolutionary or *adaptive value*, in that organisms expect to remain within their most probable (*characteristic* or *phenotypic*) states: those which make it the kind of creature that it is. In this specific sense, surprising or unexpected states (i.e., those incongruous with the expectations of the organism; e.g., a fish out of water; [15]) are deleterious, and must therefore be avoided. Hence, an organism's ultimate, *evolutionary* imperative of maintaining its repertoire of functional states within physiological bounds (i.e., survival, homeostasis, and allostasis) translates into a proximal avoidance of surprising states [15]. Following EST, this propensity to avoid surprise is the product of selection: self-organising systems that can avoid surprising phase-transitions have been favoured by natural selection over those that could not [16].

**Fig. 2:**
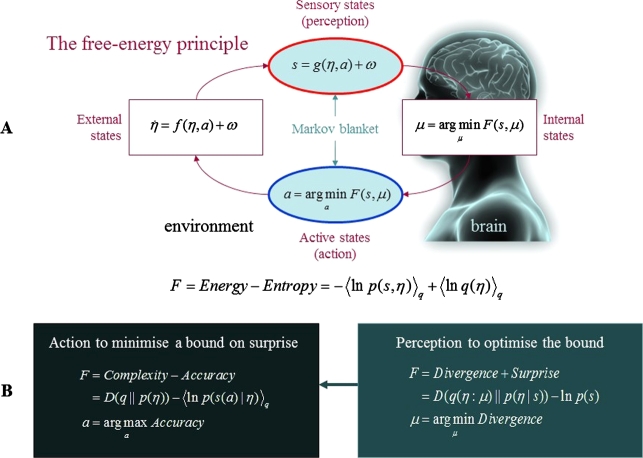
*The free-energy principle*. (**A**) The quantities that define variational free-energy. These quantities reflect a partition of the system into its internal states, *μ*, (e.g., states of the brain) and the quantities that describe its exchanges with the environment; namely, sensory input, *s* = *g*(*η*,*a*)+*ω*, and action, *a*, which alters the ways in which the organism samples its environment. The environment itself is specified by equations of motion, $\overset{˙}{\eta }=f(\eta ,a)+\omega$, which describe the dynamics of (hidden) states of the world, *η*. The term *ω* denotes random fluctuations. Both internal and active states change synergistically to minimise variational free-energy. This free-energy is a function of sensory input and a probabilistic representation of hidden environmental causes (i.e., variational density), *q*(*η*:*μ*), which is encoded by the system's internal states. (**B**): Alternative expressions for variational free-energy, which show what its minimisation entails. With respect to action, free-energy can only be suppressed by increasing the accuracy of sensory data (i.e., selectively sampling data that are predicted). Conversely, the optimisation of internal states makes the representation (i.e., variational density) an approximate conditional density over the causes of sensory input (i.e., perception, which minimises the divergence between the variational and true posterior density). This optimisation allows the variational free-energy to impose a tighter bound on surprise and enables the system to act upon the world to avoid surprising sensory and physiological states. Reproduced from [3].

So how does the FEP pertain to hierarchical neural dynamics? The FEP aligns with the theory of predictive processing by casting the brain as a hierarchically-organised ‘inference machine’ that optimises the evidence for an organism's model of the world by minimising variational free-energy (see Fig. 2). When relating the FEP to prediction and inference, the key move is to note that surprise is the negative logarithm of Bayesian model evidence. It therefore follows that any creature that minimises surprise is simply optimising Bayesian model evidence.^5^ On this view, brain dynamics (i.e., the general ‘behaviour’ or ‘ensemble dynamics’ of neural mechanisms) can be described as realising an implicit *hierarchical generative model*: a Bayesian hierarchy of ‘hypotheses’ or ‘best guesses’ about the hidden causes of our sensory states. This ‘Bayesian mechanics’ imposes an upper limit on surprise by tracking and minimising discrepancies between incoming sensory inputs and top-down, neuronally encoded predictions (i.e., *prediction errors*; [20], [31], [53], [54]). Conditional expectations are encoded by deep pyramidal cells (i.e., *representation units*) at each level of the cortical hierarchy that convey predictions downward to suppress errors at the level below; prediction errors are encoded by superficial pyramidal cells (i.e., *error units*) that convey errors forward to revise expectations at the level above; and neuromodulatory mechanisms regulate the relative influence of these signals by modifying their precision (see Fig. 1b; [31], [49], [50], [55]).

Notably, this scheme has also been leveraged to explain the functional integration of hierarchically modular neural networks. According to this view, the brain minimises prediction error by dynamically adjusting the synaptic efficiency of connections between modules, with backwards connections conveying predictions to lower levels and forward connections delivering prediction errors to higher ones [20]. Here, cognition is described in terms of the global integration of local neuronal operations via hierarchical (error minimising) message passing between cortical regions, a process that is facilitated by a hierarchically nested network architecture [20].

Under the predictive processing formulation outlined here, prediction errors quantify the organism's variational free-energy (and by extension, its surprise). This predictive process allows us to minimise surprise by updating our internal models (i.e., through perception, learning, and phenotypic plasticity). Alternatively, we can also minimise surprise by selectively sampling sensory data that confirms our expectations, to ensure that our predictions are self-fulfilling (i.e., action). Fig. 2 illustrates these two interdependent surprise-resolving processes in terms of *action*, which minimises (a *bound* on) surprise; and *perception*, which reduces the divergence between inferred and true states of the world (given some sensory data).

The ensuing perspective on brain dynamics that resolve free energy through loops of action and perception is called *active inference* [56], [57], [58]. Put simply, this suggests that action and perception operate synergistically to maintain homeostasis and optimise the organism's generative model [15], [59]. In other words, every organism seeks to maximise sensory evidence for its own existence; it is essentially ‘self-evidencing’ [60]. Quite literally, then, the FEP alludes to Maslow's [61] ‘hierarchy of needs’ – it suggests that the meaning of life is to self-actualise.

Although surprise cannot be directly evaluated by living systems, it can be minimised vicariously by minimising a bound on, or proxy for, this quantity: variational free-energy [15], [16], [52]. Because surprise is mathematically equivalent to the negative log probability of an outcome (also known as Bayesian model evidence in machine learning), minimising free-energy compels us to make Bayesian inferences about our eco-niche. Under the FEP, over time and on average, our actions will tend to infer or reflect the statistical structure of the environment to which they are coupled. This explains the *intentionality* or *purposiveness* of living systems by appealing to dynamical principles drawn from complexity science and information theory in physics, thereby providing a mathematical account of *actions guided by our beliefs*. Correspondingly, the FEP supplies a formally expressible and neurobiologically plausible physics of the mind [3], [4], [62].

An important corollary of this view is that our generative models are optimised by evolution, neurodevelopment and learning [3], [63], [64]. If we are all adapted to our own eco-niche – either through natural selection, development, or learning – then the expectations of each of us must differ. Clearly, though, some part of these expectations must also be inherited, since the characteristic, phenotypical features of a given species' generative model are conserved across generations (e.g., the basic wiring of the human brain). This segues nicely into the role of our (Bayesian) *prior beliefs* about the ways we expect the world to unfold.

By way of explanation, the FEP proffers an elegant, formally expressible explanation of human neural dynamics across spatiotemporal scales – it can be used to formulate mathematical models of the influence of natural selection acting on human phenotypes over time [3], [4], [63]. The brain only labels a sensory state as valuable or unsurprising if it leads to another valuable state, and selection ensures that an organism progresses through a succession of probable states with adaptive (homeostatic) value – intrinsic, phenotypic states that are *unsurprising* [15], [16], [52]. Under this view, natural selection reduces surprise by specifying the value of sensory states through (epi)genetic mechanisms, prescribing a small number of attractive states with innate value. These states are sought out by living systems because they minimise surprise by conforming to both their internal states and eco-niche [15]. With these distinctions in mind, species-typical patterns of cognition and behaviour can be explained as inherited *adaptive priors* that have been shaped by selection to guide action-perception cycles towards unsurprising states (e.g., “I will keep moving until I am rewarded”; [15], [52], [56]); also see [65], [66], [67]. In other words, natural selection is nature's way of performing Bayesian model selection to minimise the variational free-energy of our phenotypes (i.e., hierarchical generative models); also see [63]. The upshot of all of this is that the brain does not just *contain* a hierarchical generative model of the world, its dynamics also *instantiate* one – its form and function reflect a physical transcription of causal regularities in the environment that has been optimised by evolution within and across nested spatiotemporal scales.

Indeed, central to the architectural claim of the HMM is the evolution of hierarchical neural connections that reflect lawful statistical regularities in the environment. Take, for example, the statistical independence between the *identity* and *location* of objects in the visual world – knowing *what* an object is does not tell us *where* it is. Strikingly, this statistical independence is reflected in the anatomical dissociation between the ventral and dorsal streams in the cortical hierarchy, which encode models or representations of the ‘what’ and ‘where’ attributes of visual precepts, respectively [68]. This suggests that the structure of the brain recapitulates the structure of the world in which it is embedded: environmental causes that are statistically independent are encoded in functionally and anatomically segregated neuronal structures. Similarly, the hierarchical organisation of the brain mirrors the hierarchically nested structure of causal regularities in the environment. This hierarchical nesting marries the hierarchy of temporal scales at which representations evolve with the hierarchy of temporal scales at which biological phenomena unfold – the lower, more peripheral layers of the neural hierarchy encode rapid environmental fluctuations associated with sensorimotor processing and stochastic effects; its higher, more central layers encode increasingly slower regularities related to contextual changes [69], [70], [71], [72].

The idea that the brain instantiates a generative model based on hierarchical temporal dynamics in the environment makes intuitive sense, given that the content of our sensorium changes more rapidly than its context [73]. Moreover, the actions of an organism clearly possess a temporally nested structure (e.g., an arm movement is composed of smaller elemental movements) – it thus makes sense that the organ responsible for the evaluation and selection of actions mirrors the statistical structure of the policies to be selected. The temporal structure of neural dynamics has also been demonstrated empirically, both by simulations of perceptual inference and motor behaviour [71], [74], and studies of the human and primate brain [72], [75], [76]. Finally, there is good reason to suppose that a temporal neural hierarchy is likely to have been favoured by selection: it optimises perceptual inference by allowing the organism to accumulate evidence across different timescales to derive the best explanation for sensory data [77], and it facilitates adaptive behaviour by enabling top-down, cognitive control to achieve distal goals [78]. Other selection pressures are also likely to have been involved. For example, it has been suggested that during co-evolution with conspecifics, a temporal hierarchy would have been favoured by selection because it enables the organism to generate and recognise communicative behaviour that unfolds over multiple nested timescales [71], [73], [79]. As we discuss later, a hierarchical structure is also thought to confer other evolutionary advantages. On a more general note, it is almost self-evident that if the brain entails a generative model of (the causal structure of) its sensorium – and this sensorium is generated by deep (i.e., hierarchical) temporal processes (e.g., by conspecifics) – then neuroanatomy and neurophysiology must reflect this deep architecture. In this sense, the brain's hierarchical and modular anatomy exemplifies the good regulator theorem in cybernetics, which states that any system that can control its environment must be a good model of that environment [69], [80].

So far, then, we have described non-substantive principles that can be generalised to any species with a brain – not to mention other biological dynamics (e.g., single-celled organisms [52]; morphogenesis [81], [82]; and plant life [83]). Every species is equipped with naturally selected Bayesian priors that emerge from species-typical eco-niches and influence morphology, cognition, and behaviour in adaptive (i.e., valuable) ways – different organisms instantiate unique ‘embodied models’ of their specific biological needs and eco-niches [3], [58], [84], [85]. Nevertheless, in order to explain the *human* brain – and its relation to our cognition and behaviour – we need to draw upon substantive (*ultimate* or *evolutionary*) explanations that can account for the particular adaptive solutions that have produced the hierarchical generative models of *Homo sapiens* [31], [63], [86], [87]. With this in mind, we argue that the FEP demands recourse to psychology, because it sheds direct light on the complex, multiscale processes that govern human biobehavioural dynamics in particular. The HMM does this by synthesising the FEP with an EST of psychology.

## 3 The evolutionary systems theory of psychology

**Fig. 3:**
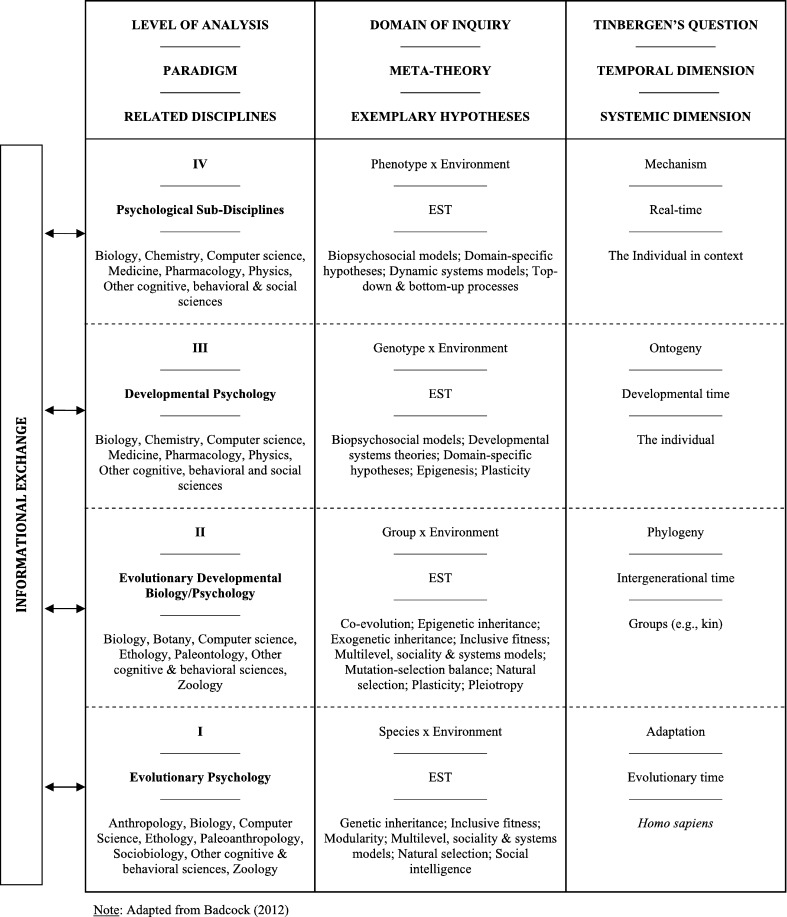
*The evolutionary systems theory of psychology*. Human phenotypes, cognition and behaviour emerge from circular interactions between (general and natural) selection and self-organisation operating within and across Tinbergen's four domains of biological dynamics (i.e., *adaptation*, *phylogeny*, *ontogeny*, and *mechanism*). The various fields of psychological inquiry explain this process by formulating models of human phenomena according to four intersecting levels of analysis: evolutionary hypotheses to explain species-typical, adaptive traits (i.e., evolutionary psychology); explanations for intergenerational, between-group differences (i.e., evolutionary developmental biology and psychology); ontogenetic explanations for individual similarities and differences (i.e., developmental psychology); and mechanistic explanations for real-time biobehavioural phenomena (i.e., the sub-disciplines). These levels of analysis are commensurate and complementary: evolutionary theories tackle the *ultimate* questions of psychology by explaining the adaptive properties of human cognition and biobehaviour; dynamic systems approaches address its *proximate* questions by shedding light on the intergenerational, developmental, and real-time mechanisms responsible for producing such phenomena. This perspective encapsulates and synthesises the various paradigms and sub-disciplines of psychology: the recursive informational exchange between different fields of inquiry allows researchers in each subfield to constrain their research in light of advances in others, and to integrate findings across different levels of psychological analysis to develop unique, substantive hypotheses. Importantly, the non-substantive meta-theory of EST, which formalises the interaction between (both *general* and *natural*) selection and self-organisation, permeates all four explanatory levels and imposes distinct inclusion criteria upon any derivative of the meta-theory itself: any multi-level hypothesis derived from this EST must conform to these two fundamental principles. Adapted from [1].

In psychology, evolutionary systems approaches have traditionally focused on the complex interplay between evolutionary and developmental processes (e.g., [88], [89], [90], [91], [92], [93], [94], [95], [96], [97]). This approach has since been developed into an integrative EST of human cognition and behaviour that has the potential to unify major paradigms in the discipline [1]. The EST in question recasts Tinbergen's [98] seminal four questions in ethology in terms of a temporal hierarchy of biological dynamics that extend across all *Homo sapiens*: those that produce species-typical ‘*functional adaptations*’ to the environment over evolutionary time (e.g., natural selection); intergenerational, ‘*phylogenetic*’ mechanisms that introduce evolutionary change by producing heritable differences between groups (e.g., epigenetic and cultural inheritance); ‘*ontogenetic*’ processes that unfold over an individual's lifetime (e.g., gene-environment interactions); and the proximate ‘*mechanisms*’ that drive psychology and behaviour in real-time (i.e., ecobiopsychosocial dynamics) (see Fig. 3).^6^ These dynamics are arguably recapitulated by different research programs in psychology, which concentrate differentially on four complementary levels of explanation: *ultimate* explanations for adaptive, species-typical traits (i.e., *evolutionary psychology*); *epigenetic* and *exogenetic* explanations for intergenerational, between-group differences (i.e., *evolutionary developmental biology* and *psychology*); *ontogenetic* explanations for individual similarities and differences (i.e., *developmental psychology*); and proximate, *mechanistic* explanations for real-time phenomena (i.e., *psychological sub-disciplines*; e.g., cognitive, social and clinical psychology) [1]. An important implication of this view is that in order to explain a given trait, one should seek to incorporate theories and evidence drawn from each of these levels of inquiry.

So how does this EST of psychology relate to the HMM? Put simply, the HMM is a *first-order hypothesis* derived from the synthesis of this broader meta-theory with the FEP – it explains the hierarchical dynamics of the embodied, situated human brain in terms of (natural and general) selection and self-organisation co-acting across evolutionary, intergenerational, developmental, and real-time spatiotemporal dynamics. The HMM is broadly consonant with other dynamical theories that cast adaptive biobehavioural patterns as the historical product of reliably recurrent developmental resources reborn in each generation. These resources are themselves the result of circular interactions between mechanisms of selection; intergenerational and developmental processes; and the engagement of humans with their species-typical environments in real-time [25], [89], [93], [102], [103], [104], [105], [106], [107], [108].^7^ By extension, the HMM suggests that the most effective way to explain the multiscale dynamics of our brains and behaviour is to develop substantive, multilevel models in psychology that are able to address both *why* certain neurocognitive and biobehavioural phenomena are adaptive in *Homo sapiens*, along with *how* these phenomena emerge from the broader causal processes that act on human phenotypes across different timescales [1], [2], [3].

There is ample evidence to suggest that this temporal hierarchy of biological dynamics manifests in the development and morphology of the brain. Indeed, both comparative work and studies on humans suggest that the evolutionary history of the human brain is reflected across nested levels of neural organisation, extending from the genes inherited from our hominid ancestors; to the epigenetic transcription factors that shape gene expression; to the synaptic epigenesis of neural networks over the course of development; through to the highly distributed and integrated long-range connections that underwrite conscious awareness [110]. Longitudinal imaging studies examining the maturation of neural networks throughout childhood and adolescence have also found that the development of the human cortex mirrors our phylogenetic history – the standard developmental sequence involves the maturation of phylogenetically older, canonical *sensorimotor hierarchies* that are common among all mammals, through to the evolutionarily recent, highly integrated *association cortices* enjoyed by humans (e.g., [111], [112], [113]). This highlights the complementarity of selection and self-organisation: natural selection ensures the emergence and retention of highly specialised or segregated sensorimotor networks in infancy, which function as ‘neurodevelopmental anchors’ that permit the progressive self-organisation of widely distributed ‘domain-general’ association regions throughout ontogeny that enhance evolvability by allowing us to respond fluidly to a constantly changing environment [25], [113], [114], [115]. Consistent with this, both evolutionary and developmental psychologists have long maintained that the brain instantiates a nested hierarchy of neuronal processing mechanisms that vary in degrees of functional segregation and integration [22], [23], [89], [90], [93], [94], [95], [116], [117], [118], [119], [120], [121], [122], [123].

Crucially, this idea is backed by extensive empirical support, ranging from large meta-analyses of neuroimaging data that provide evidence for functionally diverse ‘domain-general’ neural subsystems [124], [125], [126], through to studies of cross- and multi-modal context effects in early sensory processing that show that even at the level of the sensorium, highly segregated ‘domain-specific’ systems exchange data in a bidirectional fashion [127], [128]. On the other hand, high resolution network-based analyses have recently provided evidence that different neural ‘modules’ perform discrete cognitive functions, while highly distributed ‘connector’ regions allow for their functional integration by coordinating connectivity between ‘modules’ [129], [130]. Comparative work has further shown that a hierarchical architecture is a ubiquitous feature of the mammalian brain, progressing from highly segregated sensorimotor hierarchies found in all mammals through to the higher cortical association areas that confer the adaptive advantage of heightened cognitive control among primates [113], [115].

Of particular relevance, the brain's hierarchical organisation also resonates with EST. A hallmark feature of complex adaptive systems is that aggregates of interacting units (e.g., modules) are organised in a hierarchically nested manner; and that intra-component (e.g., within-module) connections tend to be stronger than inter-component (e.g., between-module) connections, with neighbouring components showing stronger connections than distal ones [11], [131]. As we alluded to earlier, there is broad agreement in the life sciences that this sort of structure confers significant selective advantages. First, it enhances *evolvability* because deleterious changes to single components of the system are less likely to lead to total system failure. Similarly, a hierarchical structure enables the emergence of evolutionary novelties (e.g., exaptations) without threatening global functioning [21].^8^ Spatially compact, functionally connected modules that are relatively sparsely connected to other modules also conserve the (spatial, processing, and metabolic) cost of neural connections; preserve specialised kinds of neural processing that unfold over multiple timescales; and support complex brain dynamics that optimise information processing [21], [37]. Consistent with this, fine-grained functional connectivity studies suggest that a hierarchical structure allows cortical networks to optimise the balance between local, specialised processing and global integration [129], [130]. Interestingly, computer simulations of evolving networks have also shown that even in the absence of modularity, a hierarchical structure improves evolvability by adapting faster to new environments than non-hierarchical structures, because such a structure allows the system to solve problems by recursively combining solutions to sub-problems [133].

Finally, the hierarchical organisation of the brain promotes *self-organised criticality* (colloquially, the ‘edge of chaos’; [12]). This is a fundamental property of complex adaptive systems that refers to a dynamical state that occupies the intersection between highly ordered, stable structures and cycles of activity (e.g., lattice structures); and highly stochastic, rapidly fluctuating ones (e.g., gaseous states). This state is known to optimise evolvability by allowing small, extrinsic changes to create and channel large-scale systemic reorganisations [12], [134], [135]. Recent empirical work has shown that the hierarchical segregation of neural networks into local neurocognitive mechanisms effectively stretches the parameter range for self-organised criticality [46]. The nested hierarchy of the brain means that the system can maintain different degrees of randomness, because it is able to entertain subcritical and supercritical dynamics in different modules simultaneously [136]. Given the selective advantages of being poised at the edge of chaos, it is unsurprising that a hierarchical structure, which extends this critical region, has been observed empirically [137].

In closing, it is worth noting that the picture of the brain that we have sketched above could be readily applied to all primates, not to mention other species [3], [62]. In this sense, the HMM might be forwarded as a theory of *embodied, situated brains in general*. Although we certainly encourage such efforts, the reason we have explicitly coupled this theory with paradigms in *psychology* is because they are ideally positioned to cast direct light on the highly sophisticated patterns of free-energy minimisation unique to *Homo sapiens* [87]. This is important, because it underscores the need to synthesise the FEP with substantive research that concentrates on the cognitive and behavioural dynamics particular to the species in question [4]. To address this, the HMM weds a generalisable model of the embodied, hierarchical brain with a clearly articulated *meta-theory of different levels of explanation in psychology* – which, in principle, encapsulates the disciplinary content knowledge accumulated by psychologists to date. On the one hand, this model requires psychologists to explore how the FEP applies to their own research avenues; on the other, it requires cognitive and behavioural scientists to develop – in a bottom-up, evidence-driven fashion – multilevel hypotheses about human neural and behavioural dynamics (i.e., process theories) that are substantiated by extant findings in psychology.^9^ In this sense, the HMM is as much a *heuristic for theorising and research* as it is a *theory of the brain* – an issue that we will return to shortly. Before we do, however, it is important to clearly establish how this conceptual treatment of the brain directly relates to the mathematics of the FEP. With this in mind, we will now define the HMM by leveraging the FEP to formally model the dynamics of the embodied human brain across all four levels of explanation in psychology.

## 4 The HMM defined

**Fig. 4:**
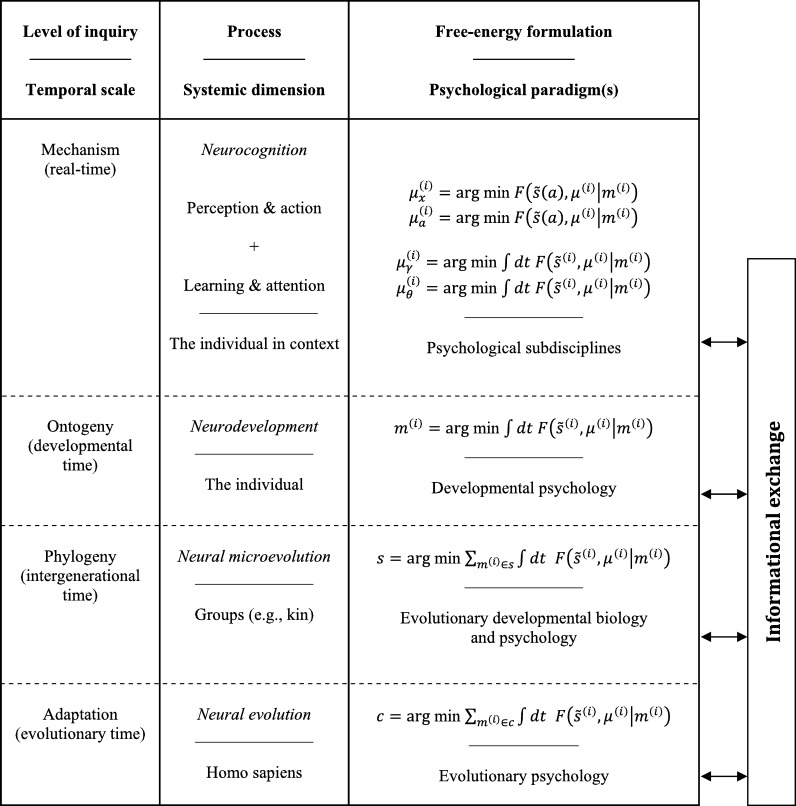
*The hierarchically mechanistic mind*. $F(\overset{˜}{s}(a),\mu^{\left(i\right)}|m^{\left(i\right)})$ denotes the variational free-energy of sensory data (and its temporal derivatives), $\overset{˜}{s}(a)$, as well as the states, *μ*, of an agent, $m^{\left(i\right)}\in s$, that belongs to a subgroup, *s* ∈ *c*, of a given class, *c*. Action, *a*, regulates the sampling of sensory data; while the internal states of the organism, *μ*, encode expectations and predictions (i.e., Bayesian beliefs) about the mean of a probability distribution. Under this formalism, *neurocognition* entails two dynamically coupled processes. The first optimises neuronal and effector dynamics (i.e., *perception* and *action*) to attune the organism to its environment – by minimising prediction errors (resp. free-energy) based on a generative model of the hidden causes of sensory data. The second process optimises synaptic strength and efficacy – over seconds to hours – to encode causal structure in the sensorium and the precision of prediction errors (i.e., *learning* and *attention*). *Neurodevelopment* optimises human generative models through activity-dependent pruning and the maintenance of neural structures and connections, which are transmitted epigenetically. *Neural microevolution* optimises average free-energy over generations of individuals belonging to a subgroup (e.g., kin) of a given class (i.e., conspecifics) via the (exo- and epi-)genetic transmission of generative models. *Neural evolution* optimises average free-energy over time and individuals of a given class (i.e., conspecifics) through the effects of selective pressure on their generative models or priors. Reproduced from [3].

Beyond the fact that they are both ESTs that explain the adaptive, hierarchical dynamics of the embodied brain, the FEP converges with the EST of psychology in two pivotal ways. First, we noted in Section 2 that although every organism is adapted to its specific eco-niche, each generation inherits the adaptive priors of the previous generation (i.e., species-typical phenotypic traits). This means that we need to consider the *systemic* dimension of these phenomena; i.e., the multiscale dynamics that extend from all *Homo sapiens* to specific individuals that operate in real-time. Second, both the FEP and EST of psychology rest on the notion of recursive, causal interactions between dynamics at different *temporal scales*. Fig. 4 shows how to express this process formally at each of the timescales over which free-energy minimisation optimises the state (i.e., *perception*), configuration (i.e., *action*), connectivity (i.e., *learning* and *attention*), anatomy (*neurodevelopment*), and phenotype (i.e., *neural evolution*) of living agents that belong to a given class (e.g., *Homo sapiens*) [3].

To recapitulate, the HMM syntheses a multi-level EST of human psychology with the variational formulation of the FEP to provide both a substantive and formally expressible theory of the brain, mind and behaviour [1], [3]. More precisely, this hypothesis defines the human brain as: *an embodied, complex adaptive control system that actively minimises the variational free-energy* (*and, implicitly, the entropy*) *of* (*far from equilibrium*) *phenotypic states via self-fulfilling action-perception cycles, which are mediated by recursive interactions between hierarchically organised* (*functionally differentiated and differentially integrated*) *neurocognitive processes*. These ‘mechanics’ instantiate *adaptive priors*, which have emerged from selection and self-organisation co-acting upon human phenotypes across different timescales. Having now defined the HMM, we will close by focusing on its implications for theorising and research.

## 5 Using the HMM as a research heuristic in neuroscience and psychology

At this juncture, we have described both a *formal* and *substantive* theory of the human brain that unifies major paradigms spanning neuroscience and psychology – one that affords both an *ultimate* (i.e., evolutionary) and *proximate* (i.e., process) theory of our cognition and behaviour (i.e., *adaptive free-energy minimisation*); and explains the psyche in terms of hierarchical neural dynamics that work vicariously to minimise surprise. This model should be seen as a *first-order hypothesis* derived from the psychological meta-theory of EST and the FEP. In other words, the HMM shares the same epistemic status as other influential theories of the brain, such as *predictive processing theory* [31], [49], [50] and the *massive modularity hypothesis* [22], [141], [142], because it provides cognitive scientists with a systematic heuristic to pose far-reaching questions and engineer unique, evidence-based hypotheses from which more specific, testable predictions can be derived [4]. Crucially, some of these predictions make the HMM amenable to falsification. In particular, the HMM relies on the directly testable *second-order hypothesis* that the brain minimises prediction error via hierarchical message passing in the brain (i.e., *predictive coding*; [31]), which has already been demonstrated experimentally by studies of visual processing (e.g., [16], [31], [55]).

For cognitive neuroscientists, the HMM encourages a range of research avenues already advocated elsewhere. First, it requires finer maps of effective neural connectivity informed by multiscale structural connectivity findings, along with empirically informed biophysical and computational models of spatiotemporal patterns of network activity that capture the ways in which our unique predictive capacities manifest in particular patterns of hierarchical neural activity across different contexts [20], [143], [144]. It also appeals to multiscale network approaches that measure neural activity across timescales, coupled with complementary methods that map rapid fluctuations in neural patterns in real-time (e.g., EEG and MEG), maturational changes over developmental time (e.g., diffusion tensor imaging), and neural mechanisms conserved by evolution (e.g., comparative studies) [138], [145], [146], [147]. Finally, the HMM resonates with approaches in *embodied cognition* and *neuroethology*, which both explore how action-perception cycles emerge from adaptive brain-body-environment relations [3], [4], [148], [149], [150], [151]. Collectively, these approaches suggest that our understanding of hierarchical neural dynamics depends on the cumulative weight of empirical methods that are differentially suited for diverse, synergistic ends.

More generally, the HMM forges a dialectical relationship between neuroscience and psychology – it favours mutual enlightenment and cross-fertilization between both of these disciplines by allowing insights gleaned from the one to inform and constrain theorising and research in the other [116], [152], [153], [154]. For neuroscientists, this requires approaches that can isolate the specific *psychological factors* that govern different patterns of hierarchical neural activity across different contexts. Methodologies conducive to this kind of research include meta-analyses of task-based fMRI activation studies to characterise the functional fingerprints of particular neural regions across different tasks [124], [125], along with empirical work on the development of ‘cognitive ontologies’ that systematically map the distinct relationships between well-defined cognitive functions and particular patterns of neural dynamics [155], [156]. Furthermore, methods from developmental psychology can allow neuroscientists to better evaluate the ways in which different developmental trajectories lead to distinctive styles of behaviour and temperament (which, under the FEP, correspond to different kinds of error-minimising policies, which can vary a great deal between individuals). In this vein, longitudinal designs that combine neuroimaging studies on human brain maturation with carefully chosen biological, psychological and social measures might be used to explore how different developmental contexts engender stable individual differences in perceptual biases and active inference [157]. Comparative, cross-cultural, computational, and dynamical approaches stemming from evolutionary psychology also allow us to study in great detail the (epi)genetic mechanisms responsible for the emergence, transmission, and acquisition of our species-typical adaptive priors [138], [152]. Finally, computational models and simulation studies enable us to model how different levels of dynamical activity interact [3], [149], [158], [159], [160], allowing neuroscientists to explore how the biobehavioural phenomena described and studied by psychologists reflect adaptive free-energy minimisation within and across different spatiotemporal scales. The outcomes of such analyses can then be confirmed through real-world observations and experimental work [4], [138].

On the other hand, the FEP offers both a biologically plausible and empirically tractable *formal theory* of the human brain, mind, and behaviour to psychologists. Traditionally, proponents have relied chiefly on computer simulations, fMRI and EEG to apply dynamic causal models of interactions between hierarchically organised cortical areas in order to explain perception (e.g., [79]), action (e.g., [57]), attention (e.g., [161]), and learning (e.g., [162]). More recently, though, others have taken up the FEP to explain a wide range of psychological phenomena (see [49], [50]), including anxiety [163], autism [164], [165], [166], emotion [167], [168], [169], meta-cognition [170], and both self- and other-representations [171], [172]. It also lends itself to methods that are highly familiar to psychologists, such as the P300 – an event-related potential that may be used experimentally as a non-invasive, temporally sensitive proxy for surprise [173], [174]. As discussed elsewhere, the FEP can also be exploited to explain large-scale sociocultural phenomena, including the hierarchical dynamics of scientific theorising itself [1], [3], [87], [140], [175].

Indeed, by incorporating the FEP, the HMM proffers a new way to explain cognition and behaviour that can be readily applied to all levels of psychological inquiry [1]. Although the mathematical apparatus that underwrites it may seem inaccessible, active inference can be reduced to a simple rubric that can be leveraged by researchers across psychology's sub-disciplines – *cognition and behaviour function together to minimise surprise*. In other words, our lives are a self-fulfilling prophecy of sorts: everything we think and do stems from the biological imperative to optimise our predictions about causal regularities in our eco-niche, and to behave in ways that confirm them. Like others before us (e.g., [31], [50], [60], [163], [166]), we believe this elegant idea offers a common language to synthesise and explain diverse findings across the discipline. More particularly, the HMM calls for integrative hypotheses rallied around four complementary research questions: *What, if any, is the adaptive function of the phenomenon in question? What are the evolutionary, intergenerational, developmental, and real-time mechanisms that produce it? How does it exemplify active inference? And how does it emerge from particular patterns of hierarchical neural dynamics?*

**Fig. 5:**
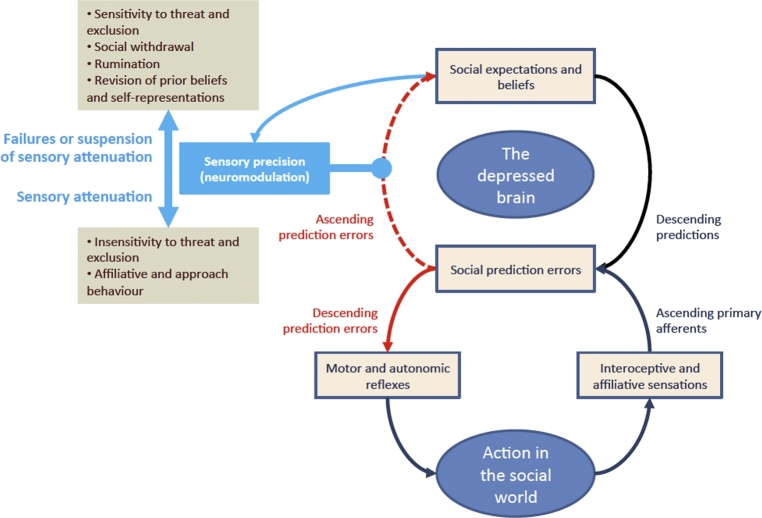
*The evolutionary systems model of depression*. Under active inference, motor and autonomic reflexes mediate action and are driven by descending (*proprioceptive* and *interoceptive*) prediction errors (e.g., reflexes that resolve sensory prediction errors). Action entails the attenuation of ascending prediction errors (i.e., the down-regulation of precision). Prediction errors cannot always be resolved through action; in which case, the attenuation of sensory precision is suspended. This suspension enables ascending prediction errors to revise posterior beliefs, which improves the accuracy of top-down predictions. Here we apply active inference to depressed mood states. Under this model, when depression is *adaptive*, it engenders an increase in the precision of (bottom-up) social (interoceptive and affiliative) prediction errors when an individual is faced with the threat of aversive interpersonal outcomes (e.g., exclusion). This increased precision improves perceptual inference and learning about the probable causes of social stimuli: it heightens sensitivity and directs attention to socio-environmental cues, while reducing confidence in (top-down) social predictions. Cognitively, this is reflected by the inhibition or suspension of goal directed behaviour (e.g., anhedonia), along with an attentional bias toward social cues and increased rumination about self-other relations. However, depression becomes pathological when there is a pervasive failure of sensory attenuation, which induces aberrant beliefs about the likelihood of social rewards and engenders negative expectations about interactions with others (e.g., pessimism, low self-esteem). These expectations of negative social outcomes can become self-fulfilling, because they can lead the individual to search for sensory evidence that social rewards are improbable and suppress exploratory or acquisitive interpersonal behaviours (i.e., those with uncertain outcomes). Behaviourally, both adaptive and pathological depressed states reduce uncertainty within the social world by down-regulating reward-approach behaviours (e.g., anhedonia, social withdrawal), and by generating signalling behaviours that elicit interpersonal support (e.g., reassurance seeking) and defuse potential conflict (e.g., submissive behaviours). Reproduced from [2].

As we mentioned at the outset, this modelling approach has already been used to develop an evidence-based EST of the human capacity for depression. Combining previous applications of the FEP with research spanning all four levels of explanation in psychology, this *second-order hypothesis* suggests that depression typically reflects an evolved biobehavioural strategy that responds adaptively to noxious social conditions (e.g., exclusion) by minimising the likelihood of unpredictable interpersonal exchanges. According to this view, normative depressed mood states instantiate a *risk-averse adaptive prior* that reduces the likelihood of deleterious social outcomes by causing adaptive changes in perception (e.g., heightened sensitivity to social risks) and action (e.g., risk-averse interpersonal behaviours) when sensory cues indicate a high degree of socio-environmental volatility ([2]; see Fig. 5). As discussed elsewhere, this is a neurobiologically plausible scheme that has important implications for diagnosis and treatment in clinical psychology, which can also be leveraged by cognitive and behavioural scientists to derive more specific, testable predictions [2].

## 6 Conclusion

In this article, we have proposed an EST of the human brain predicated on neuroscience and psychology alike. Although we believe that the HMM offers a unifying theory of the brain, cognition and behaviour that has the potential to benefit both of these disciplines by demanding their integration, its explanatory power clearly rests on the cumulative weight of the second-order hypotheses and empirical evidence that it generates [4], [138]. Naturally, whether our model inspires consequential research remains to be seen. If it does, however, it will require sophisticated, collaborative efforts to elucidate how dynamical interactions between evolutionary, intergenerational, developmental and real-time processes govern particular patterns of cognition and biobehaviour; the ways in which the FEP explains such phenomena; and the hierarchical neural dynamics responsible for producing it. Although developing such multilevel models is fraught with complexity, a desire for simplicity should not obstruct our pursuit of veracity. Ultimately, the Devil dwells in the details.

## Footnotes

1 EST is a transdisciplinary paradigm that hearkens from [5] Schrödinger E. What is life? Cambridge: Cambridge University Press; 1944. EST explains all adaptive dynamic systems in terms of circular interactions between *self-organisation* and *general selection* (e.g., *natural selection*) within and across (hierarchically nested) spatiotemporal scales [1] Badcock PB. Evolutionary systems theory: a unifying meta-theory of psychological science. Review of General Psychology. 2012;16:10–23, [3] Ramstead MJD, Badcock PB, Friston KJ. Answering Schrödinger's question: A free-energy formulation. Physics of Life Reviews. 2018;24:1–16. This universal, dynamical process creates *complex adaptive systems*, which adapt to the environment through an autonomous process of selection that recruits the outcomes of locally interacting components within that system to select a subset of those components for replication or enhancement [6] Levin S. Complex adaptive systems: exploring the known, the unknown and the unknowable. Bulletin of the American Mathematical Society. 2003;40:3–19. Of particular relevance here, a widely cited example is the brain [7] Haken H. Principles of brain functioning: a synergetic approach to brain activity, behaviour and cognition. Berlin: Springer-Verlag; 1996, [8] Kelso JS. Dynamic patterns: the self-organization of brain and behavior. Cambridge, MA: MIT Press; 1995. For in-depth discussions of the reciprocally causal relationship between selection and self-organisation, see [9] Depew DJ, Weber BH. Darwinism evolving: systems dynamics and the genealogy of natural selection. Cambridge, MA: MIT Press; 1995, [10] Eigen M, Schuster P. The hypercycle: a principle of natural self-organisation. Berlin: Springer-Verlag; 1979, [11] Holland JH. Hidden order: how adaptation builds complexity. New York: Basic Books; 1995, [12] Kauffman SA. The origins of order: self-organization and selection in evolution. Oxford: Oxford University Press; 1993. For a succinct treatment of the underlying mathematics, also see [13] Ao P. Emerging of stochastic dynamical equalities and steady state thermodynamics from Darwinian dynamics. Communications in Theoretical Physics. 2008;49.

2 In network neuroscience, this kind of subsystem is called a *module*[21] Sporns O, Betzel RF. Modular brain networks. Annual Review of Psychology. 2016;67:613–40. We have avoided the term here because of its potential confusion with *massive modularity*, *domain-specificity*, and *informational encapsulation*[22] Barrett HC, Kurzban R. Modularity in cognition: framing the debate. Psychological Review. 2006;113:628. [23] Barrett HC. A hierarchical model of the evolution of human brain specializations. Proceedings of the National Academy of Sciences. 2012;109:10733–40. [24] Fodor JA. The modularity of mind: An essay on faculty psychology. Cambridge, MA: MIT press; 1983. For incisive critiques of the use of ‘modularity’ in psychology, see [25] Anderson ML, Finlay BL. Allocating structure to function: the strong links between neuroplasticity and natural selection. Frontiers in Human Neuroscience. 2014;7, [26] Chiappe D, Gardner R. The modularity debate in evolutionary psychology. Theory & Psychology. 2012;22:669–82, [27] Colombo M. Moving forward (and beyond) the modularity debate: A network perspective. Philosophy of Science. 2013;80:356–77, [28] Frankenhuis WE, Ploeger A. Evolutionary psychology versus Fodor: Arguments for and against the massive modularity hypothesis. Philosophical Psychology. 2007;20:687–710, [29] Zerilli J. Against the “system” module. Philosophical Psychology. 2017;30:235–50.

3 Note that the levels of organisation listed here are a gross approximation. A hierarchically nested organisation is likely to produce different topologies at different neural scales, and the precise, hierarchical organisation of neural elements at different levels remains open to question [48] Hilgetag CC, Goulas A. Is the brain really a small-world network? Brain Structure and Function. 2016;221:2361–6., [45] Kaiser M. A tutorial in connectome analysis: topological and spatial features of brain networks. Neuroimage. 2011;57:892–907.

4 Please note an important caveat about the relationship (and major differences) between the variational formulation described here and statistical thermodynamics: variational free-energy should not be conflated with thermodynamic free-energy. The free-energy formulation is a mathematical description of the dynamics of systems at *nonequilibrium steady-state*, and should not be confused with the second law of thermodynamics. The FEP deals with information theoretic measures (e.g., variational free-energy, mutual information, relative entropy, self-information, surprisal, information gain, Bayesian surprise, Shannon entropy, *etc*.). The connection between the variational free-energy for nonequilibrium steady-state systems and thermodynamic free-energy in statistical thermodynamics remains an outstanding question. For more details, see [4] Ramstead MJ, Badcock PB, Friston KJ. Variational neuroethology: Answering further questions: Reply to comments on “Answering Schrödinger's question: A free-energy formulation”. Physics of Life Reviews. 2018;24:59–66. For an account of this connection by appeal to Landauer's principle and the Jarzynski equality, consider [51] Sengupta B, Stemmler MB, Friston KJ. Information and efficiency in the nervous system—a synthesis. PLoS Computational Biology. 2013;9:e1003157.

5 Technically, variational free energy provides an upper bound on surprise or self information, via the addition of a nonnegative Kullback–Leibler divergence to surprise that induces an approximate posterior density over the causes of sensory states. This means that the negative free-energy lower bounds log evidence. In this sense, it is sometimes referred to as an *evidence lower bound* (ELBO) in machine learning and statistics. It also means that minimising surprise effectively means minimising the variational free-energy via an approximate posterior encoded by the brain's internal states. See Fig. 2.

6 It should be recognised that this is only one interpretation of Tinbergen's questions, which continue to attract debate. For example, in this context, phylogeny is used to refer to the intergenerational *processes* responsible for producing evolutionary change within a species, not the evolutionary *outcomes* of such processes (e.g., our species' position on a phylogenetic tree). There is also considerable debate about whether Tinbergen's ‘survival value’ should be equated with ‘adaptation’, ‘function’ or ‘current utility’ [99] Bateson P, Laland KN. Tinbergen's four questions: an appreciation and an update. Trends in Ecology & Evolution. 2013;28:712–8, [100] Bateson P, Laland KN. On current utility and adaptive significance: a response to Nesse. Trends in Ecology & Evolution. 2013;28:682–3, [101] Nesse RM. Tinbergen's four questions, organized: a response to Bateson and Laland. Trends in Ecology & Evolution. 2013;28:681–2. We have settled upon ‘functional adaptation’ here because of its clear evolutionary connotations. As we have discussed elsewhere, how one applies Tinbergen's questions will also vary depending on the systemic and temporal scales under scrutiny [3] Ramstead MJD, Badcock PB, Friston KJ. Answering Schrödinger's question: A free-energy formulation. Physics of Life Reviews. 2018;24:1–16, [4] Ramstead MJ, Badcock PB, Friston KJ. Variational neuroethology: Answering further questions: Reply to comments on “Answering Schrödinger's question: A free-energy formulation”. Physics of Life Reviews. 2018;24:59–66, [62] Ramstead MJ, Constant A, Badcock PB, Friston K. Variational ecology and the physics of minds. Physics of Life Reviews. Accepted, this issue. Leaving such terminological issues aside for the purposes of the current discussion, it suffices to say that human phenotypes emerge from the dynamic interplay between evolutionary, intergenerational, developmental, and real-time causal processes, which are differentially illuminated by major paradigms in psychology.

7 Also note that a particularly important constraint that extends across all of these timescales is the sociocultural environment, since human survival depends on our ability to leverage cultural information and immersively participate in normative, culturally adapted practices [3] Ramstead MJD, Badcock PB, Friston KJ. Answering Schrödinger's question: A free-energy formulation. Physics of Life Reviews. 2018;24:1–16, [87] Ramstead MJ, Veissière SP, Kirmayer LJ. Cultural affordances: scaffolding local worlds through shared intentionality and regimes of attention. Frontiers in Psychology. 2016;7, [109] Gallagher S. Enactivist interventions: Rethinking the mind, Oxford: Oxford University Press; 2017.

8 As noted by a reviewer, these explanations have also been leveraged to argue that evolution favours *redundancy* in neural networks. Although we agree that redundancy is important, as discussed here, a nested hierarchy also confers additional evolutionary advantages. We would also note that it is important to distinguish between redundancy, where the same function is performed by different neuronal structures; and *degeneracy*, which entails many-to-one structure-function relationships. Specifically, while degeneracy facilitates evolution by conferring systemic robustness, the brain will seek to minimise redundancy over the course of development because it negatively affects efficiency; e.g., due to increased metabolic and other costs [132] Friston KJ, Price CJ. Degeneracy and redundancy in cognitive anatomy. Trends in Cognitive Sciences. 2003;7:151–2.

9 This is not to say, of course, that the HMM should be restricted to psychology alone – unpacking the complexities of the human phenotype clearly requires inter-disciplinary efforts, particularly those within the cognitive and social sciences, along with comparative and phylogenetic approaches [138] Daunizeau J. A plea for “variational neuroethology”: Comment on “Answering Schrödinger's question: A free-energy formulation” by MJ Desormeau Ramstead et al. Physics of Life Reviews. 2018, [139] Kirmayer LJ. Ontologies of life: From thermodynamics to teleonomics. Comment on “Answering Schrödinger's question: A free-energy formulation” by Maxwell James Désormeau Ramstead et al. Physics of Life Reviews. 2018, [140] Veissière S. Cultural Markov blankets? Mind the other minds gap!: Comment on “Answering Schrödinger's question: A free-energy formulation” by Maxwell James Désormeau Ramstead et al. Physics of Life Reviews. 2018.
